# Portomesenteric Venous Thrombosis Presenting as Bowel Gangrene

**DOI:** 10.7759/cureus.76410

**Published:** 2024-12-26

**Authors:** Sania Khalifa, Pramod Nichat, Ami S Gandhi, Viraj Gorhe, Nishit Patel

**Affiliations:** 1 General Surgery, Grant Government Medical College and Sir JJ Group of Hospitals, Mumbai, IND; 2 General Surgery, Grant Government Medical College and JJ Group of Hospitals, Mumbai, IND

**Keywords:** bowel gangrene, intestinal obstruction, pancreatitis, portal vein thrombosis, superior mesenteric thrombosis

## Abstract

Portal vein thrombosis (PVT) typically arises in patients with underlying cirrhosis, hepatobiliary malignancies, abdominal inflammatory conditions, or hematologic disorders. However, in non-cirrhotic individuals, PVT is less common and may initially present with minimal symptoms, escalating significantly if it extends to the mesenteric veins. Here, we present the case of a 37-year-old male with combined portal and mesenteric venous thrombosis, manifesting as acute intestinal obstruction. He was successfully managed with an exploratory laparotomy, resection of gangrenous bowel, and systemic anticoagulation therapy. This case highlights the critical role of early surgical intervention in preventing mortality associated with non-cirrhotic PVT.

## Introduction

Acute mesenteric ischemia (AMI) results from an abrupt reduction in blood flow to the intestines and is categorized based on the affected. While mesenteric venous thrombosis is rare, its progression to bowel gangrene can be devastating if not swiftly managed. This case illustrates the critical importance of early intervention. Patients with mesenteric venous thrombosis typically present in two stages: an initial phase marked by abdominal pain that is disproportionate to physical examination findings, followed by ischemic progression that can lead to intestinal stricture and obstruction [1]. The four main types are superior mesenteric artery (SMA) embolism, accounting for approximately 50% of cases; atherosclerotic SMA occlusion or thrombosis, comprising 15-25% ; non-occlusive mesenteric ischemia (NOMI) (20%); and mesenteric venous thrombosis (5-15%) [1].

Mesenteric venous thrombosis is categorized by the time course of symptoms: acute (sudden onset), subacute (developing over days to weeks), or chronic. Patients with subacute and chronic thrombosis often present asymptomatically or with signs of portal hypertension-related bleeding, while acute cases without collateral formation present urgently with abdominal pain and require timely diagnosis and intervention to avoid complications [2]. 

Portal vein thrombosis (PVT) involves a blood clot in the portal vein, which carries blood from the intestines to the liver. PVT can lead to life-threatening complications like bowel gangrene if untreated and early diagnosis is critical to prevent severe outcomes. Extrahepatic portal systemic venous thrombosis (EPSVT) is a known complication of chronic pancreatitis that can involve the mesenteric, splenic, and/or portal veins. The pathophysiology of EPSVT includes local inflammatory and prothrombotic changes, inherited thrombophilic factors, and vascular compression by the pancreas. In chronic pancreatitis, these local factors often play a more significant role in EPSVT development than thrombophilia [3]. 

## Case presentation

A 37-year-old male presented with a one-month history of abdominal pain, progressive abdominal distension, and a three-day history of non-passage of stools and flatus. The pain, initially confined to the upper abdomen, was described as insidious in onset, progressively worsening, and dull in nature. Over the course of the past year, the patient had experienced three similar episodes of abdominal pain, for which intravenous analgesics provided temporary relief. Additionally, he reported nausea and three episodes of non-bilious vomiting, containing food particles, but denied fever, jaundice, or significant weight loss. There were no reports of bleeding per rectum or hematemesis.

The patient’s medical history was significant for chronic alcohol consumption (180 ml alcohol, 4-5 days per week) for 20 years and tobacco use for 8 years. On examination, the patient’s pulse was 110/min, and blood pressure was 130/70 mm Hg. Abdominal examination revealed moderate distension, mild epigastric tenderness, generalized guarding, and sluggish bowel sounds. Key diagnostic indicators included progressive abdominal distension, persistent epigastric pain disproportionate to the clinical examination, and non-passage of stools. Per rectal examination showed a collapsed rectum, no stools, and blood-stained gloves.

Table 1 presents the laboratory investigations of the patient.

**Table 1:** Laboratory investigations

Laboratory investigation	Patient value	Normal range
Hemoglobin	9.5	13-17 g/dl
Total leukocyte count	14,700	4-10,000/ul
Platelet count	669000	150-410000/ul
C-reactive protein	37	<5 mg/dl
Lipase	798	upto 190 U/L
Amylase	399	less than 86 IU/L
Serum Sodium	139	135-148 mEq/L
Serum Potassium	4.2	3.5 - 5.5 mEq/L
Prothrombin time	12.4	9.6-11.7 secs
International Normalised Ratio	1.2	0.9-1.1
Serum Creatinine	0.79	0.5-1.4 mg/dl
Serum Urea	15	10-39 mg/dl

Imaging

Radiological Image 1 presents the X-ray of the patient's abdomen showing features of small bowel obstruction.

**Figure 1:**
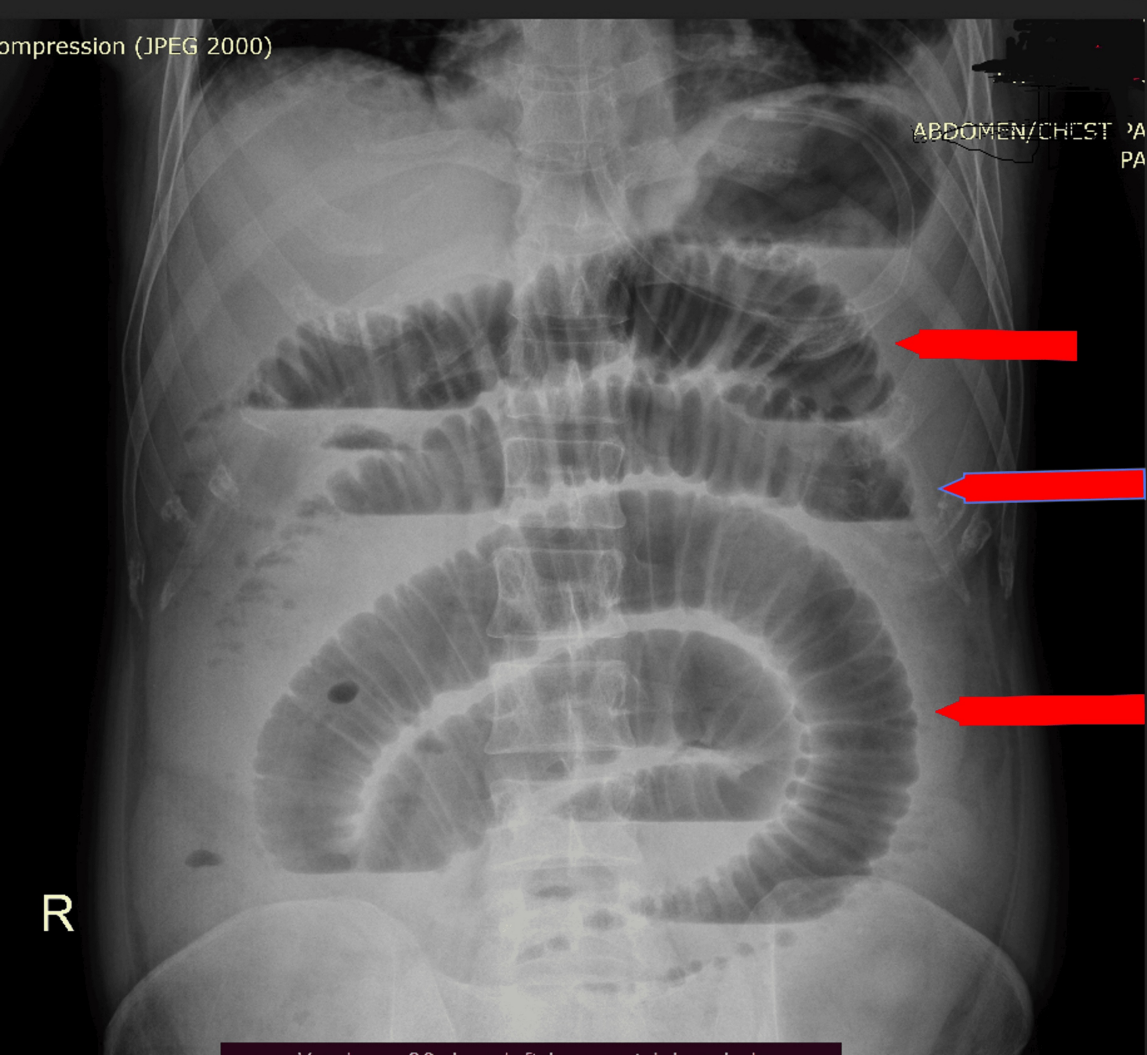
X-ray abdomen with arrows pointing at small bowel dilated air fluid levels.

Video 1 shows CT abdomen (on admission) of the patient with portomesenteric thrombosis.

**Video 1:** Contrast-enhanced CT abdomen (on admission ) in the patient presenting with portomesenteric thrombosis. Overall features of portal hypertension show thrombus occluding 90-95% of the superior mesenteric vein with extensive bowel dilation indicative of ischemia.

Table 2 shows contrast-enhanced CT (CECT) abdomen (on admission) features suggestive of mesenteric ischemia secondary to venous occlusion and features of portal hypertension.

**Table 2:** CECT abdomen findings (on admission) shows features of mesenteric ischemia secondary to venous occlusion, changes of portal hypertension. SMA: superior mesenteric artery; IMA: inferior mesenteric artery; CECT: contrast-enhanced CT

Feature	Findings	Significance
Portal Vein	Partial lumen occlusion (50-60%) with thrombus along its entire length.	Suggests portal venous thrombosis, commonly associated with portal hypertension.
Superior Mesenteric Vein	90-95% occlusion by thrombus	Critical indicator of mesenteric venous thrombosis (MVT)
Splenic Vein	Complete lumen occlusion.	Implicates involvement of splanchnic venous circulation, collaterals, extensive collaterals suggesting cavernoma formation indicates chronic portal hypertension.
Jejunal/Ileal Loops	Dilated loops (max diameter: 4.3 cm) with air-fluid levels.	Reflects bowel stasis and ischemia. Wall Thickening , Ileal wall thickening (3.1 mm) with hyperattenuation (+60 HU) suggestive of intramural hemorrhage.
Target Sign	Seen in some ileal loops, indicating bowel wall edema.	Characteristic sign of ischemia or inflammation.
Free Fluid	Noted in the abdomen and pelvis, especially along ileal loops.	Suggests bowel ischemia and associated inflammatory response.
Mesenteric Fat Stranding	Associated with vessel engorgement.	Supports mesenteric ischemia and venous congestion.
Pancreas	Atrophic body without parenchymal calcifications; normal duct.	Likely unrelated to acute pancreatitis.
Arteries (Celiac/SMA/IMA)	Normal	Rules out arterial occlusion as a cause of ischemia.

Repeat CECT Abdomen (48 Hours Later)

The portal vein and SMV (superior mesenteric vein) showed continued partial occlusion with thrombus, extended to the distal SMV branches. The small bowel showed increased dilation (max diameter 4.8 cm), intramural hemorrhage, and the appearance of pneumatosis intestinalis. Some segments of the ileum showed bowel wall edema and thickening (up to 7.2 mm). The overall features were suggestive of the progression of mesenteric ischemia, with worsening bowel obstruction and hemorrhagic changes.

Video 2 shows the CECT abdomen (after 48 hrs) in the patient with portomesenteric thrombosis.

**Video 2:** Contrast-enhanced CT abdomen (48 hours later) in a patient with portomesenteric thrombosis

Management

Given the clinical and imaging findings, the patient was started on intravenous heparin (5000 IU) and monitored closely. Despite this, the patient developed persistent tachycardia, decreased urine output, and worsening abdominal pain with generalized guarding, prompting an emergent exploratory laparotomy.

Intraoperative findings

The jejunal and ileal loops were dilated, friable, and congested. Around 100 cm, the segment of the gangrenous bowel (distal jejunum and ileum) was resected, and a double-barrel jejunoileostomy was created. Around 200 ml of serosanguinous fluid was found in the abdominal cavity. Other abdominal organs were within the normal limits.

**Figure 2:**
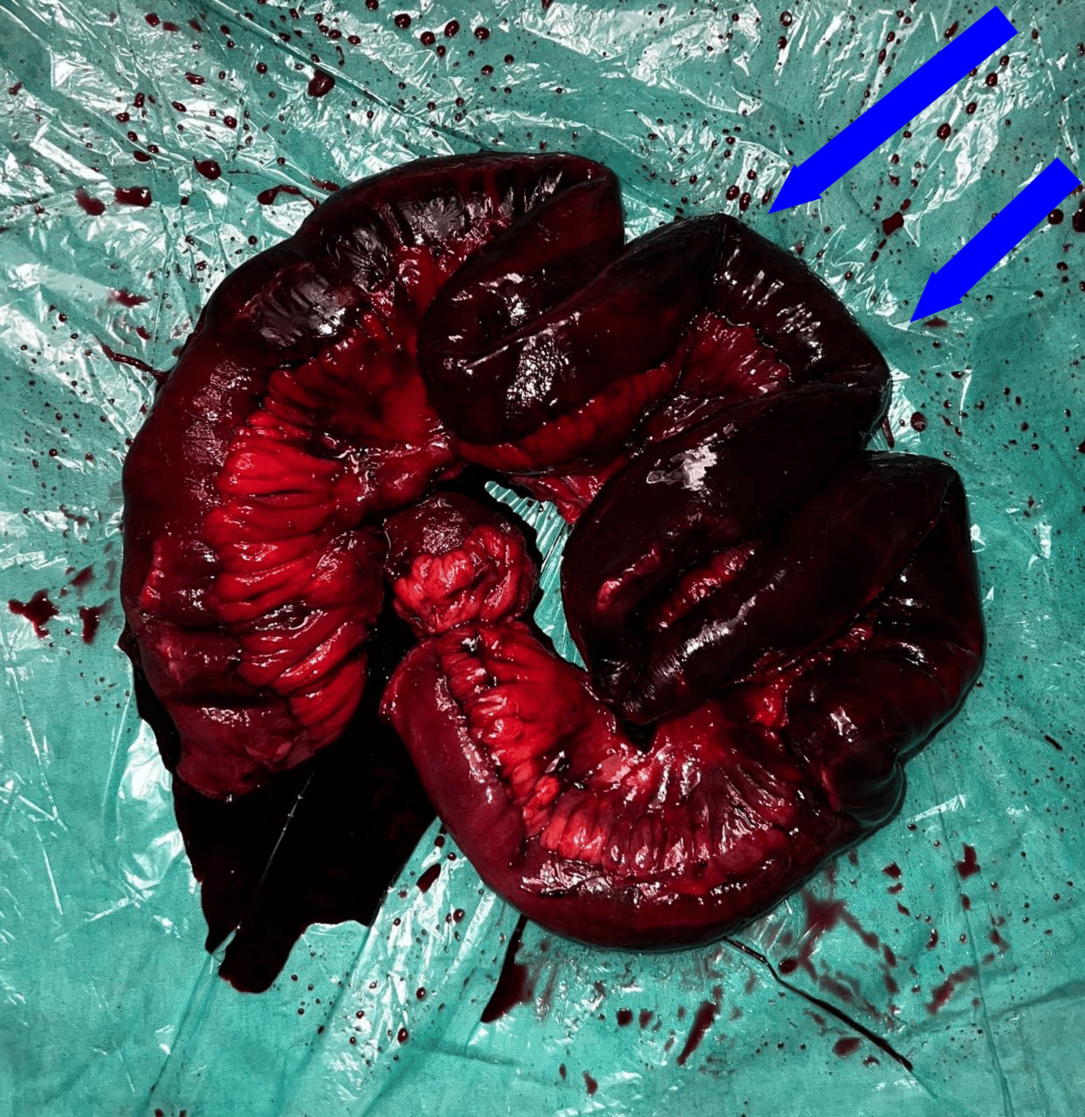
Intraoperative image showing gangrenous bowel segment (blue arrow shown )

Figure 2 shows the intraoperative image of the gangrenous bowel segment.

Postoperative care

The patient recovered uneventfully post-surgery and was started on dabigatran after overlapping with heparin for six months. He was also taught stoma care. A gastroenterology consult was requested due to signs of portal hypertension, and an EGD (esophagogastroduodenoscopy) revealed portal gastropathy with dilated fundal channels. The patient was started on lasilactone and propranolol for portal hypertension management.

Postoperatively, the patient was started on a combination of spironolactone and furosemide (lasilactone) to manage portal hypertension by reducing fluid overload and controlling ascites. Propranolol was initiated for primary prophylaxis against variceal bleeding, a known complication of portal hypertension. These interventions align with the established management strategies for portal hypertension, aiming to decrease the risk of life-threatening complications.

Follow-up

Video 3 shows the CECT abdomen after three months of follow-up.

**Video 3:** Follow-up CECT abdomen in the case of portomesenteric thrombosis after 3 months.

At three months follow-up, the patient underwent a CT distal loop cologram to evaluate for stoma reversal. The findings included the following: the Portal Vein was narrowed with distal SMV involvement and extensive venous collaterals, consistent with chronic thrombosis and cavernous transformation. Mild splenomegaly was noted. The contrast from the stoma reached the rectum, with no evidence of a leak at the suture site.

Stoma reversal was successfully performed and the patient continued regular follow-up for the management of portal hypertension.

This case highlights the complexity of mesenteric ischemia secondary to venous occlusion, particularly in patients with chronic alcohol use, and underscores the importance of early diagnosis and intervention to prevent bowel necrosis and other complications.

## Discussion

Mesenteric venous thrombosis (MVT) associated with immediate bowel infarction typically involves thrombi in the intramural vessels, arcuate veins, and vasa recta, rather than in the larger venous trunks. In contrast, thrombi in the larger splanchnic veins, which spread peripherally, are linked to a longer duration of symptoms and infarction that occurs several days later. In the acute form of MVT, when there is complete venous obstruction without collaterals, the bowel initially becomes engorged with blood, cyanotic, and eventually suffers from mucosal ischemia and transmural infarction. Concurrent arterial vasospasm further contributes to the ischemia. In the subacute form, recovery is possible due to the development of collateral circulation, but ischemia persists due to venous occlusion. Some patients may transition from the acute form to chronic MVT, in which intermittent abdominal pain occurs due to increased demand for blood supply or the development of an acute-on-chronic thrombus. MVT is most commonly seen in individuals aged 40-60 years, with a higher prevalence in males. In the acute form, symptoms typically include abdominal pain that is out of proportion to clinical examination findings, while the chronic form is associated with features of portal hypertension. As ischemia worsens, abdominal tenderness and distention become evident, and severe cases may present with guarding and rigidity [2,4]

Correlating laboratory investigations and imaging findings with clinical scenario

The patient presented with a hemoglobin level of 9.5 g/dL, consistent with mild anemia. This finding is not uncommon in individuals with chronic alcohol use, as it may result from factors such as nutritional deficiencies, gastrointestinal blood loss, or bone marrow suppression. Although the anemia was initially mild, the clinical situation necessitated the transfusion of two units of blood: one intraoperatively and another postoperatively. These transfusions were administered to optimise the patient’s oxygen-carrying capacity and ensure hemodynamic stability during and after the surgical intervention. Anemia in the context of mesenteric venous thrombosis can exacerbate tissue hypoxia, especially in cases of bowel ischemia and infarction. Adequate correction of haemoglobin levels through transfusion is critical in such scenarios to minimise further ischemia damage and improve patient outcomes [5]. The total leukocyte count is elevated at 14,700/µL, which could be indicative of an ongoing inflammatory or infectious process.

The patient’s elevated lipase (798 U/L) and amylase (399 IU/L) levels are indicative of pancreatic involvement, potentially pointing to underlying pancreatitis. While the imaging report described the pancreas as having an atrophic body, it did not provide clear evidence of acute pancreatitis. Elevated pancreatic enzymes in the setting of mesenteric venous thrombosis (MVT) warrant careful consideration, as pancreatitis is a well-documented risk factor for MVT. Pancreatic inflammation can lead to local vessel injury, increased thrombogenicity, and subsequent venous thrombosis [6]. In this case, the elevated enzyme levels, combined with the imaging findings, suggest a chronic pancreatic process rather than acute pancreatitis. However, the possibility of subclinical or resolving pancreatitis contributing to the thrombotic event cannot be ruled out. Further evaluation of the enzyme trends and a more detailed assessment of the pancreas might help clarify its role in the development of MVT. This emphasizes the importance of recognizing pancreatic involvement as a potential etiological factor in such cases.

The CECT of the abdomen revealed thrombosis of the portal vein and superior mesenteric vein, accompanied by bowel wall edema, intramural hemorrhage, and pneumatosis intestinalis. These findings are hallmark indicators of MVT with associated bowel ischemia and are consistent with progressive ischemic injury. Pneumatosis intestinalis, in particular, is indicative of severe ischemia and impending bowel necrosis [7].** **Intraoperatively, the findings of gangrenous bowel necessitating resection and stoma creation confirmed the transition from ischemia to irreversible bowel damage, as anticipated from the imaging. While the CT findings did not indicate bowel perforation, they were consistent with advanced ischemia, leading to transmural necrosis, which was evident intraoperatively. This correlation underscores the utility of imaging in predicting the severity and progression of mesenteric ischemia, facilitating timely surgical intervention to manage life-threatening complications.

MVT can arise due to several factors. Localized factors causing vessel wall injury include inflammatory conditions such as pancreatitis, inflammatory bowel disease, diverticulitis, peritonitis, and appendicitis. Thrombophilia, whether inherited or acquired, is another significant contributor. Inherited forms include deficiencies in Protein C or S, Factor V Leiden, and antithrombin, while acquired causes are hematologic conditions like polycythemia vera, antiphospholipid syndrome, and paroxysmal nocturnal hemoglobinuria, as well as non-hematologic conditions such as malignancy, oral contraceptive use, pregnancy, nephrotic syndrome, and hyperhomocysteinemia. Additional causes include intra-abdominal surgery, trauma, and conditions leading to venous stasis, such as congestive splenomegaly, cirrhosis, and congestive heart failure. Despite these known causes, a subset of cases remains idiopathic. Among intra-abdominal inflammatory processes, pancreatitis is the most common, leading to portal vein thrombosis in 3-5% of cases [4].

In the present case, thrombophilia screening was not performed due to resource limitations. However, the patient’s history of chronic alcohol use may have contributed to a prothrombotic state through liver dysfunction and portal hypertension. While no inherited thrombophilia was identified, the case could be classified as secondary to alcohol-related portal hypertension and pancreatitis. This highlights the importance of assessing both inherited and acquired prothrombotic conditions in patients presenting with MVT.

Chronic alcohol use is a recognized risk factor for thrombosis, particularly in the setting of mesenteric venous thrombosis (MVT), due to its association with both systemic and local prothrombotic states. Chronic alcohol consumption can lead to liver dysfunction, which alters the balance of procoagulant and anticoagulant factors, thereby predisposing individuals to a hypercoagulable state. Additionally, alcohol-induced portal hypertension increases venous stasis, further elevating the risk of thrombosis. Another critical link is alcohol-induced pancreatitis, which contributes to localized inflammatory processes in the peripancreatic and mesenteric regions. Inflammation can damage the vessel wall, activate platelets, and promote clot formation within the mesenteric veins [8]. In the present case, the patient’s history of chronic alcohol use likely played a multifaceted role in predisposing to MVT through a combination of liver dysfunction, portal hypertension, and potential episodes of subclinical or recurrent pancreatitis. This unique interplay underscores the importance of considering alcohol use as a potential contributory factor in patients presenting with thrombosis, even in the absence of overt pancreatitis.

Diagnosing MVT requires a high index of suspicion based on clinical history and findings. Contrast-enhanced computed tomography (CECT) is the diagnostic modality of choice, with an accuracy of 90%. It typically reveals findings such as the presence of thrombi in the mesenteric veins, bowel ischemia with bowel wall thickening exceeding 3 mm, hypoenhancement of the bowel wall, ascites, collaterals, and splenomegaly. Differentiating chronic MVT from acute MVT is facilitated by the presence of collateral circulation. Portal vein thrombosis, which may present with features of portal hypertension including splenomegaly, can also lead to portal hypertensive cholangiopathy, characterised by the formation of collateral vessels around the bile ducts [9].

In the present case, imaging findings of cavernoma formation and splenomegaly are consistent with chronic portal hypertension. Portal gastropathy observed on EGD supports this diagnosis, reflecting increased portal venous pressure and its effects on the gastrointestinal tract. Cavernoma formation, representing collateral vessel development, is a hallmark of chronic portal vein thrombosis [10]. The absence of thrombocytopenia suggests that hypersplenism was not significant in this case, but the gastropathy underscores the clinical relevance of portal hypertension in the patient’s presentation.

The treatment of MVT varies based on the severity of the condition. In cases involving venous gangrene or peritonitis, surgical management becomes necessary. Exploratory laparotomy is performed, with bowel resection aiming to conserve as much viable bowel as possible. For extensive bowel involvement, a second-look laparotomy may be required. Thrombectomy is feasible when the thrombus is localized to the superior mesenteric vein (SMV), but diffuse thrombi often preclude this option. On the other hand, medical management is suitable for cases without signs of bowel ischemia. Unfractionated heparin is the first-line treatment due to its short half-life, reversibility, and established ability to increase survival and reduce recurrence. Following stabilization, anticoagulation therapy with warfarin should commence and continue for a duration of six months [4]. The duration of therapy varies according to the aetiology as MVT caused by coagulation disorders would require lifelong anticoagulation [11].

The exact percentage of mesenteric venous thrombosis (MVT) cases requiring exploratory laparotomy is not frequently specified in studies, as surgical intervention is typically reserved for severe cases, such as those involving bowel gangrene or peritonitis. A study by Kumar et al. (2003) found that approximately 10-15% of MVT cases necessitate surgical management, while the majority are managed effectively with anticoagulation [12].

 In the present case, the patient underwent emergency exploratory laparotomy with gangrenous bowel resection and stoma creation. The patient demonstrated gradual improvement in portal hypertension markers, including reduced ascites and improved liver function tests over six months of follow-up. A repeat Doppler ultrasound confirmed decreased portal venous pressure. The patient’s stoma functioned adequately postoperatively, with no signs of obstruction or infection. The patient received education on stoma care and reported satisfactory adaptation. Bowel continuity was successfully restored after three months. Post-reversal, the patient reported significant improvement in quality of life, including a return to normal diet and activities. There were no complaints of bowel dysfunction or abdominal pain at the six-month follow-up.

## Conclusions

In conclusion, mesenteric venous thrombosis (MVT) is a significant condition with diverse causes, including inflammatory diseases, malignancies, and thrombophilic disorders. Pancreatitis, especially chronic and alcoholic forms, is a recognized cause of MVT, where inflammation rather than intrinsic thrombophilia plays a key role in thrombus formation. Early detection of MVT, primarily through contrast-enhanced CT, is vital for differentiating acute from chronic forms and guiding treatment. Management typically involves anticoagulation, with surgery reserved for cases of bowel infarction or peritonitis. Timely intervention improves outcomes, especially when combined with a multidisciplinary approach involving gastroenterologists, radiologists, and surgeons. For patients with portal hypertension, endoscopic treatment and long-term anticoagulation may be necessary.

In essence, prompt diagnosis and appropriate management are crucial to reducing complications and improving prognosis in patients with MVT.
